# Treatment and cost of pressure injury stage III or IV in four patients with spinal cord injury: the Basel Decubitus Concept

**DOI:** 10.1038/s41394-019-0173-0

**Published:** 2019-03-15

**Authors:** Christine Meier, Stefan Boes, Armin Gemperli, Hans Peter Gmünder, Kamran Koligi, Stefan Metzger, Dirk J. Schaefer, Klaus Schmitt, Wolfram Schwegmann, Reto Wettstein, Anke Scheel-Sailer

**Affiliations:** 1 Swiss Paraplegic Centre (SPC), Nottwil, Switzerland; 2 Department of Health Sciences and Health Policy, University of Lucerne, Lucerne, Switzerland; 3 Swiss Paraplegic Research (SPF), Nottwil, Switzerland; 4 Swiss Department of Plastic, Reconstructive, Aesthetic and Hand Surgery, University Hospital, Basel, Switzerland

**Keywords:** Outcomes research, Rehabilitation, Rehabilitation

## Abstract

**Study design:**

Retrospective chart analyses as part of a quality improvement project.

**Objectives:**

To demonstrate treatment of pressure injury (PI) in patients with spinal cord injuries (SCI) and analyse costs using the “modified Basel Decubitus Concept”.

**Setting:**

Inpatient setting of a specialised acute care and rehabilitation clinic for SCI.

**Methods:**

Complex treatment courses of four patients with chronic SCI and PI stage III or IV were described and costs were recorded. The total healthcare services’ costs per patient and different profession’s involvement were analysed in relation to patient characteristics, treatment phases and milestones demonstrated.

**Results:**

The treatment of PI stage III and IV in patients with SCI included input from plastic surgery, rehabilitation medicine, nursing and other involved professions. Recommended interventions were chosen according to the “modified Basel Decubitus Concept”. The cost course of PI treatment in patients with SCI depicted the multimodal treatment concept, including three clinically and financially relevant milestones (debridement, flap surgery and mobilisation to wheelchair) as well as the highest costs in the functionally highly dependent patient. Acute care and rehabilitation overlapped with different intensities during the whole treatment process.

**Conclusion:**

Multimodal treatment concepts connecting acute and rehabilitation care were applied in these complex health conditions. Cost-explication models including treatment phases and milestones helped to understand resources more easily and integrate aspects of process-based management and quality of care. Scientific evidence is needed to create a recommended quality standard in line with adequate financing of this health condition.

## Introduction

Pressure injury (PI) stage III or IV in patients with spinal cord injury (SCI) is a health condition that involves complex treatment concepts with multi-layered aligned involvement of different disciplines and professions. The treatment of PI involves cross-sectoral treatment aspects of acute and rehabilitation care [1–6]. It is the second most frequent complication [7] in individuals living with SCI and generates the highest costs among all SCI complications [8, 9].

PIs are classified according to the National Pressure Ulcer Advisory Panel in six degrees of severity (°III = full thickness tissue loss, °IV = full thickness tissue loss with exposed bone, tendon or muscle) [10]. With an increasing knowledge about the physiology and risk profiles in the development of PI stage III or IV [10–12] and short and long-term effects of different medical and surgical treatments [13], recommendations including different treatment elements came up [2, 5, 6, 14]. These concepts include preconditioning of the PI with pressure relief, highly specialised surgery procedures with different flap techniques and complex inter-professional interventions including nursing, nutrition and psychotherapy, physiotherapy and occupational therapy [15–17].

For optimising complex treatment courses to guarantee quality, clinical treatment pathways are increasingly recommended [18]. The implementation of clinical pathways should lower complication rates, shorten lengths of stay and reduce resource-use without adverse effect on patient outcome [18]. Detailed analyses of these complex treatment interventions are often missing and understanding between quality assurance, health professions and economists is low. To understand the emerging cost of a treatment course, the origin of costs in such a complex treatment concept needs to be explained. Criteria of a clinical pathway [18], such as treatment phases and milestones, might improve the transparency from a clinical perspective and translate it into cost terms and quality. In addition, information on patient characteristics, such as diagnoses and functioning according to the bio-psycho-social model [19], might explain the complexity and costs even more.

The aim of this study is to describe patient characteristics, interventions and costs in the complex treatment course of four patients with SCI and PI stage III or IV during their inpatient stay.

## Methods

### Design

We performed a case study as part of a larger quality improvement project with the following propositions, hypotheses and expectations:

- Through the demonstration of phases and milestones in a continuous process of PI stage III or IV care, treatment principles could be used for the development of quality assurance.
- The application of clinical treatment pathways connects acute and rehabilitation care.
- The analysis of the treatment processes and costs improves the understanding from a clinical and economic point of view. This understanding assures confidence of clinicians, economists, society and affected patients.

### Study setting

This research was conducted at an acute and rehabilitation clinic specialised in the care of patients with SCI. The clinic is committed to a process-based corporate development. As part of a continuous quality improvement specialised clinic, inter-professional quality circles are responsible for best clinical practice by defining standards of care and implementing quality controlling as in the case of PI treatment.

#### The modified Basel Decubitus Concept

The treatment of PI stage III or IV in patients with SCI follows the six treatment principles of the “modified Basel Decubitus Concept” [1, 5, 14, 20, 21].

Luscher and colleagues [20, 21] first described the “Basel Decubitus Concept” for PI stage III or IV in patients with SCI in 1992, including (1) Pressure relief, (2) Debridement and infection control, (3) Wound conditioning, (4) Treatment of risk factors and nutritional optimisation, (5) Flap surgery, and (6) Prevention of secondary complication and education.

Based on years of experience, this concept was modified and an international collaboration re-evaluated the different components of the “Basel Decubitus Concept” in several consensus conferences. Although single aspects are evidence based, the concept in its complexity has never been proven.

**Fig. 1:**
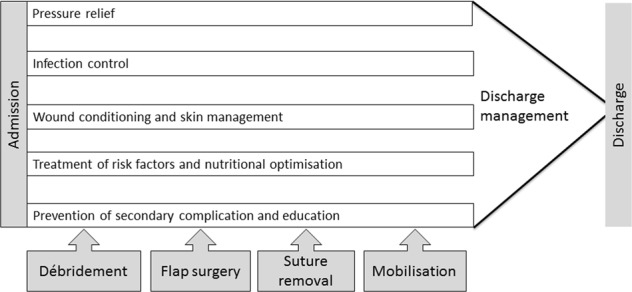
Interdisciplinary pressure injury treatment concept of the Swiss Paraplegic Centre, modified after Scheel-Sailer et al. [14]

The modified “Basel Decubitus Concept” includes the following relevant single intervention components and relevant milestones displayed schematically in Fig. 1 and described in detail in Table 1.

**Table 1:** Detailed description of the modified “Basel Decubitus Concept”

Detailed description of the modified “Basel Decubitus Concept”
Classification and documentation
Classification of PI according to the international classification of PI defined by the EPUAP [10, 33] and NPUAP Documentation following international recommendations including photos [34, 35].
Pressure relief
Immediately immobilisation after admission for inpatient treatment, either on airflow mattresses or if possible with supine ventral positioning [25, 36, 37].
Wound conditioning
A local dressing according to the TIME concept [25, 34]. Necrosis to be removed and treatment of infection whenever possible only with local disinfection. Surgical debridement to accelerate the cleaning phase. Wet dressings three times per day or negative pressure wound therapy [36, 38].
Treatment of risk factors and nutritional optimisation
Risk screening during this cleaning phase: general internal medical aspects as well as specific SCI risk factors. *Standard laboratory* examinations focussed on examination of infection, anaemia, electrolytes, kidney and liver function and nutrition profile [36, 39]. Administration of the Spinal Cord Nutrition Tool (SNST) to detect nutrition deficiencies. Nutrition counselling, to address the special needs during the treatment of the PI, recognise the changed protein and calorie requirements [39]. Examination of *pulmonary functioning* (pulmonary function test) and breathing therapy (inhalation and physiotherapy) risk reduction of pneumonia during immobilisation [40, 41]. *Cardiovascular diagnostics*: electrocardiogram and thoracic radiography (initiated if clinically indicated) [14]. Examination of the lower extremities and the spine: standardised *range of motion* (e.g. contraction, scoliosis) as a risk factor in the seating position. *Neurological examination*: in case of change with the international standard for neurological examination in SCI (ISNCSCI) [42]. Screening of *psychological risk factors*: psychological consultation and integrated psychotherapy if an individual indication is confirmed, focussing on risk behaviour, coping strategies and the psychological consequences of immobilisation [24, 25, 43, 44].
Surgical treatment: Debridement and flap surgery
Flap surgery performed through Plastic surgeons. Closure of PI with tension free fascio-cutane tissue without scars over bony prominences if possible. Established flap techniques for different regions: os ischium—posterior thigh flap, os sacrum/coccygis—gluteal rotation flap, os trochanter—tensor fasciae latae flap [5, 36]. Diagnosis of osteomyelitis: performed through bone biopsies during the surgical debridement or during flap surgery. Infection treatment with specified antibiotics, duration approximately 6–8 weeks [36]. According to the diagnoses of osteomyelitis, postsurgical bed rest for 4–6 weeks [14].
Prevention of secondary complications and education
Prevention of secondary complications resulting from the interdisciplinary risk analyses and the individualised therapeutic process. Re-evaluation of the seating position and the seating cushions. Re-evaluation of transfer techniques and strength training of the upper extremities. Local functional electrical stimulation [45, 46]. Evaluation of the outpatient environment including auxiliaries and need of nursing support. In cases of work reintegration, evaluation of the work load

*EPUAP* European Pressure Ulcer Advisory Panel, *NPUAP* National Pressure Ulcer Advisory Panel, *PI* pressure injury, *SCI* spinal cord injury

### Study population

Patients with an SCI, who were admitted for flap surgery of PI of stages III or IV, located sacral, trochanteric or ischial, between March 2016 and March 2017, were included in the sampling. The patients were selected by convenience sampling balanced for different characteristics.

### Data collection

#### Data collection of patient and treatment characteristics

Patient data were collected retrospectively from a patient’s chart review according to recommended standards [22], including gender, age, lesion characteristics, PI characteristics, treatment milestones, complications, antibiotic treatment for osteomyelitis and length of stay.

#### Collocation of health service-related costs

Healthcare service cost data were summed up in different disciplines and profession-specific categories (Table 2).

**Table 2:** Classification of disciplines

Group of disciplines	Included health service cost
Costs related to plastic surgery	Costs of materials^a^, drugs^a^ and services^b^ of plastic surgeons, surgery ward and anaesthesiology
Physical and rehabilitation medical costs	Charges for time^c^ of the medical doctors spent with and for the patient on the ward
Nursing costs	Material^a^, drugs^a^ and services^b^ of nursing personnel at the intensive care unit; costs for nursing time^c^ spent with/for the patient on the ward
Therapies and counselling costs	Costs of therapy services^b^ of occupational therapy, physical and physio therapy, psychology, nutritional therapy, speech therapy, and social counselling; costs of materials^a^ used in occupational therapy or prepared for use at home
Additional medical consultations costs	Costs of materials^a^, drugs^a^ or services^b^ of inpatient pain service, neuro-urology, radiology, respicare, orthopaedics, interventional medicine, hand surgery and inpatient use of outpatient services
Ward costs	Costs of bandage^a^ and other material^a^ on the ward; laboratory services^b^ performed exclusively on the ward; additional general services^a^ applied on the ward by nurses (only nurse services not recorded in nursing minutes)
Laboratory costs	Costs of internal^b^ and external^a^ laboratory services
Medicament costs	Costs of drugs^a,d^ provided on the ward by nursing personnel

^a^Total purchase price

^b^Integrated tariff model (ITAR_K) (Hplus, 2017, Internet) calculation

^c^Cost rate per minute [47]

^d^Purchase price per unit

Health service-related data were collected from the central service management of the hospital that provided a main data set (plastic surgery-related costs, nursing, therapies and counselling, ward and laboratory costs) (Table 2). In addition, daily prescriptions of special medication and daily time in minutes (hours and minutes) of physical and rehabilitation medicine physicians were documented manually.

All service data was grouped into patient weeks constructed in 7-day steps from admission onwards over the whole inpatient stay. Data were controlled for plausibility and corrected if necessary by the research team.

Health services were recorded in different units in the clinic, as tax points, time units, number of services or monetary unit. The cost calculation into monetary units was performed through specific calculations. The complete costs were compared to the patient cost rates of the clinic, which are calculated for each patient by the finance department.

## Results

### Case description

Patient characteristics are described in Table 3. None of the patients developed a complication related to PIs after flap surgery, such as delayed wound healing or similar. Patient (P) 2 showed the highest functional dependency. P1 and P2 had a cervical SCI. P1 was the only patient not suffering from osteomyelitis. P4 was early-discharged 5 days after the first-ever mobilisation in wheelchair after treatment.

**Table 3:** Patient characteristics

	Patient 1	Patient 2	Patient 3	Patient 4
Gender	Male	Female	Female	Male
Age, years	38	56	29	56
Aetiology of SCI	Traumatic	Traumatic	Congenital	Traumatic
Level of lesion	C7	C5	Th11	Th5
Completeness of lesion	AIS A	AIS A	AIS A	AIS A
SCIM admission	24	17	38	59
Relevant secondary diagnosis	Suprapubic catheter, traumatic brain injury, spasticity	Spasticity, smoker, diabetes mellitus	Anxiety disorder	Multiple pressure injury on feet, spasticity, colostomy, peripheral artery occlusive disease (PAOD)
Pressure injury stage [14]	4	4	4	4
Localisation of pressure injury	Sacral bone	Sacral bone	Sacral bone	Ischium
Osteomyelitis	No	Yes	Yes	Yes
Complication related to pressure injury	No	No	No	No
Inpatient stay (days)	79	72	92	55
Antibiotic treatment (days)	47	56	47	56
Immobilisation (weeks)	4	6	6	6

*AIS* American Spinal Injury Association Impairment Score, *SCI* spinal cord injury

All patients followed the usual treatment including all milestones and treatment phases. P3 represented the longest inpatient stay with 92 days compared to the shortest inpatient stay of 55 days (P4). P2–4 were immobilised for a period of 6 weeks due to osteomyelitis leading to an antibiotic treatment for 6 weeks (Table 3).

### Particular healthcare services' costs

**Fig. 2:**
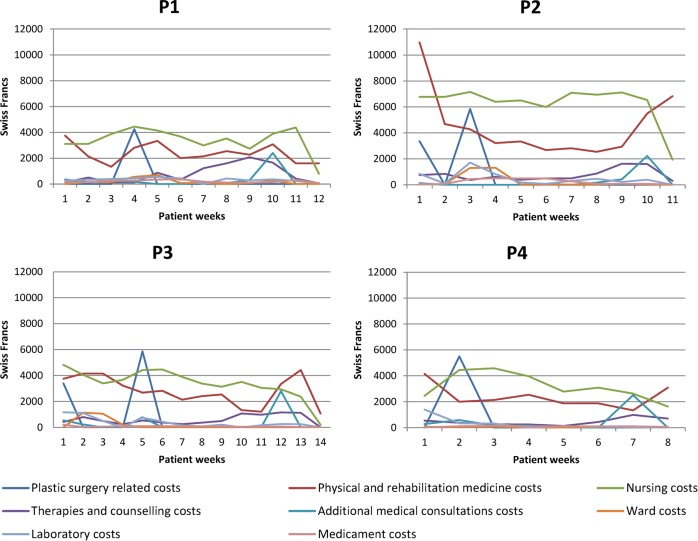
Healthcare services’ cost course per patient (P) 1–4

The particular healthcare services’ costs of the grouped professions of all patients during the inpatient stay are illustrated in Fig. 2. All patients’ treatment costs corresponded to the proposed continuous treatment phases. Three of the four milestones were depicted in the cost courses of professions. Debridement, flap surgery as well as the increasing therapy and counselling costs around the wheelchair mobilisation build up cost peaks. All patients showed continuously and predominantly high nursing costs compared with the costs of other professions. Within the first 5 weeks, the peaks in laboratory costs were explained by the investigation of peri-surgical diagnostics, infection control and nutritional status in P2–4. The slightly higher costs of medication of P2 illustrated more expensive antibiotic medication of this patient. All patients indicated an increase of additional medical consultations costs in the last 2–4 weeks of their inpatient stay. For most of the therein-comprised interventions, a change of body position is required, justifying why these interventions were not effectuated earlier in the treatment process during strict immobilisation. The health service cost calculation and the cost calculated by the finance department of all four patients did not differ.

### Total healthcare services' costs

**Fig. 3:**
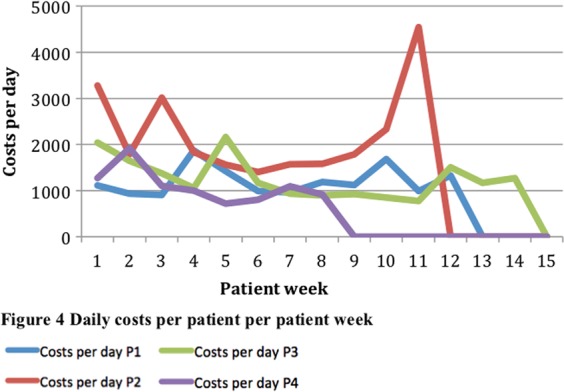
Daily costs per patient per patient week

The course of total health services’ scaled costs per week is illustrated in Fig. 3 for all patients. Scaled costs represented the plastic surgical milestones of the treatment in all patients by clear cost peaks in the respective weeks (P1: week (W) 1, P2: W1 and W3, P3: W1 and W5, P4: W2). On suture removal, all patients showed a decrease of costs compared to the previous week. For the wheelchair mobilisation, P1, P2 and P4 showed an increase of day costs compared to the previous 2 weeks. The scaled costs of P3 increased after the first week of mobilisation.

The cost courses of the included patients were found to reflect the treatment course (Figs. 2 and 3). Clear congruence was found for two of four clinical treatment milestones as well as the treatment phases with slightly lower costs during the phase before mobilisation (Figs. 2 and 3). While the plastic surgery interventions were financial relevant milestones, suture removal was not found to reflect its clinical relevance as a milestone in financial terms. Mobilisation in wheelchair in this sample did partially support its financial relevance as a milestone despite its clinical relevance for the patient (Figs. 2 and 3).

## Discussion

The cost course assured the implementation of the “modified Basel Decubitus Concept,” demonstrating the intensity of the different therapies during the treatment phases. The treatment of PI stage III and IV in patients with SCI included interventions from plastic surgery, rehabilitation medicine, nursing and other involved professions. The cost course of PI treatment in patients with SCI depicted three clinically and financially relevant milestones (debridement, flap surgery and mobilisation to wheelchair) as well as the highest costs in the functionally highly dependent patient.

### Patient data and health service-related data

This study investigated data of four patients with SCI and PI stage IV at the most frequent localisations of PI sacral and ischial with a higher percentage of PI with osteomyelitis (30% compared to 75% in our population) [5]. Length of stay in our patients with 55–92 days was within the range of a retrospective observational cohort study [2] and shorter than the average length of stay in previous research with an average of 98 days [5]. However, the research of Wettstein et al. included 47% PI of the ischial region, estimated to be related to prolonged wound healing and therefore longer length of stay [2, 5]. In the present research, only P4 had an ischial PI.

Both the shorter length of stay and the absence of PI-associated complications lead to the assumption that in the present samples the most expensive patients with PI and SCI are not included. Among the included patients, no PI-associated complication occurred, contrasting with previous research. Complication rates in this health condition range usually between 30% and 50% [2] and 35% for non-SCI PI patients [23]. Complication rates are related to the length of stay [2] and simultaneously to the overall costs [2, 5]. This fact does not influence the main topic of the study to demonstrate congruence of therapeutic intervention and cost course but it has to be taken into account when talking about quality.

### Process-based treatment concepts and costs

Patients passed through all the stages of PI treatment according to the treatment process [1, 14]. The method of detailed analyses in this four purposively selected patients clearly confirmed exemplarily the implementation of the “modified Basel Decubitus Concept” [1] including a complex interdisciplinary and inter-professional treatment process.

The plastic surgery interventions play an important role in the treatment of the PI stages III and IV from a clinical perspective [1, 14] and triggered relevant costs as shown from the economic perspective. The surgical and anaesthesiologist procedures not only include many different treatment processes and a quality assurance that is not part of this level of analyses but also explains the cost intensive period. Also the wound conditioning and pressure relief between a debridement and flap surgery or even before debridement had a bearing on the treatment and financial process indicated by initially high nursing materials and airflow bed-systems [1, 14]. The time-consuming, specialised wound care and the complex care during complete immobilisation explained the nursing costs of the included patients, mainly in the pre- and early post-surgical phase. A constant presence of specialised health professionals during all phases was shown and confirmed the relevance [2, 14].

Post-flap immobilisation prohibits different interventions, which include the active participation or mobilisation of the patient to achieve best tissue healing at the level of different layers [1, 6]. Thus many examinations are not allowed during this period and reflected in slightly lower costs during this period compared with the phases of admission or discharge indicated by higher costs of different additional examination such as e.g. urodynamics. These examinations are usually part of regular annual SCI controls and the organisation of these preventive measures during the mobilisation phase express a patient-centred care to reduce organisational burden compared to a later outpatient planning. However, the support of health professionals was inevitable during the immobilisation phase to prevent complications related to the PI or complications provoked by the immobilisation such as frequent complications like pneumonia [2] or internistic health conditions such as anaemia in combination with SCI and PI.

The suture removal during the post-flap immobilisation phase triggered an increased number of therapeutic interventions, for example, suture massages, strengthening of upper extremities and stretching lower extremities the week before mobilisation in the wheelchair. The expected increase in costs could not be calculated by the presented data. Therapists adopt their intervention to the intended goal and can use the intervention time either for prevention as respiratory training or transfer training, therefore an increase of costs did not appear.

The “modified Basel Decubitus Concept” also includes transfer training, evaluation of seating position, initiating functional electrical stimulation and education with an integrated individualised psychotherapy as secondary prevention to reduce recurrence rates usually being between 20% and 50% during the first year and later on [2, 24, 25]. These activities explain the ongoing high costs of therapy intervention in the last phase of the treatment.

The permanently high remaining nursing costs during the whole treatment process were explained by low patient functioning related to tetraplegia. P2 with the highest functional dependency of the included patients caused the highest costs. Nursing services were previously found to be the most prevailing service provided to neurologic patients [26] and inducing one of the highest costs among neurologic inpatient services [27]. Higher average per-day nursing time minutes than in previous data [28] are possibly explained by the higher functional dependence of the included four patients during immobilisation phase, thus functioning information is crucial to achieve adequate cost transparency already during acute treatment phases [29].

Regarding costs and quality, the middle- and long-term effect of complex intervention is crucial. The concept aims at reducing complication and relapse rates, as well as related sorrow and costs. We exemplarily showed that process-based management is also applicable in complex health intervention as part of a quality assurance programme. The combined principles of process-based management, evidence and consensus-based standard might give the fundamental information for reimbursement according to quality assurance programmes [18, 30].

Although long-term evidence to reduce recurrence for these interventions does not yet exist, a discussion concerning the need of these interventions as part of a quality assurance or reduction of complex intervention treatments to reduce costs is still needed. First guideline development activities concerning this topic came up and supported the implementation of best practice. A structured consensus process to agree on a standard and a prospective cohort study in different specialised centres might help to increase knowledge in this complex field even more.

### Implications

Incentives of reimbursement models and casemix systems are in discussion in many healthcare systems. As the present study supports the higher costs provoked by a higher functional dependency and the relevance of functioning information for casemix systems [9, 29], further studies need to model the effect of functioning information also for rare but cost-intensive health conditions such as PI in SCI. An in-depth understanding of the effect of functioning on costs will assure care for these patients in the future.

Process-based treatment concepts including patient relevant characteristics as baseline, components of inter-professional treatment and milestones as well as standardised documentation of complications can build the foundation for transparent reimbursement models [18]. An international guideline including best evidence and highly specialised experts might provide confidence from clinical and economic sides to guarantee best quality of care in this complex health condition.

### Limitations

This research treats data of four cases and analyses the data deeply. Therefore, the results have exploratory character and do not allow for conclusions in a larger sample of patients with SCI and PI.

In service data collection, the sharpness of different variables varied and some data might be missing despite the data plausibility and quality control due to the complexity of collecting service data and their respective costs. The individually collected patient-related costs do not fully cover the real complete costs in the hospital. Some general hospital costs cannot be allocated to the individual patient.

## Conclusion

The cost course of PI treatment in patients with SCI reflects the treatment phases and three of the four milestones as well as highest costs in the functionally highly dependent patient. The presented data on costs therefore improve the coincident understanding of the clinical and financial aspects of the treatment process. Multimodal treatment concepts connecting acute and rehabilitation care need to be favoured in complex health conditions. Clearly, this research supports the consideration of treatment phases and milestones of a complex treatment concept.

## Data archiving

Original data are stored at the clinical trial unit of the clinic.
