# Correction: Treatment and cost of pressure injury stage III or IV in four patients with spinal cord injury: the Basel Decubitus Concept

**DOI:** 10.1038/s41394-019-0184-x

**Published:** 2019-05-08

**Authors:** Christine Meier, Stefan Boes, Armin Gemperli, Hans Peter Gmünder, Kamran Koligi, Stefan Metzger, Dirk J. Schaefer, Klaus Schmitt, Wolfram Schwegmann, Reto Wettstein, Anke Scheel-Sailer

**Affiliations:** 1 Swiss Paraplegic Centre (SPC), Nottwil, Switzerland; 2 Department of Health Sciences and Health Policy, University of Lucerne, Lucerne, Switzerland; 3 Swiss Paraplegic Research (SPF), Nottwil, Switzerland; 4 Swiss Department of Plastic, Reconstructive, Aesthetic and Hand Surgery, University Hospital, Basel, Switzerland

**Keywords:** Outcomes research, Rehabilitation, Rehabilitation

**Correction to: Spinal Cord Series and Cases** 5:30

10.1038/s41394-019-0173-0

published online 15 March 2019

This article was originally published under standard licence, but has now been made available under a [CC BY 4.0] license. The PDF and HTML versions of the paper have been modified accordingly.

